# Pain Control during the Treatment of Primary Palmar Hyperhidrosis with Botulinum Toxin A by a Topical Application of Liposomal Lidocaine: Clinical Effectiveness

**DOI:** 10.3390/toxins16010028

**Published:** 2024-01-06

**Authors:** Andrea Marani, Helena Gioacchini, Matteo Paolinelli, Ivan Bobyr, Emanuela Martina, Giulia Radi, Federico Diotalallevi, Anna Campanati

**Affiliations:** 1 Clinic of Dermatology, Department of Clinical and Molecular Sciences, Polytechnic University of Marche, 60126 Ancona, Italy; S1102257@pm.univpm.it (A.M.); S1103456@pm.univpm.it (H.G.); ivan.bobyr@ospedaliriuniti.marche.it (I.B.); emanuela.martina@ospedaliriuniti.marche.it (E.M.); Giulia.radi@ospedaliriuniti.marche.it (G.R.); federico.diotallevi@pm.univpm.it (F.D.); 2 Dermatology Unit, “Infermi” Hospital of Rimini, 47900 Rimini, Italy; matteo.paolinelli@auslromagna.it

**Keywords:** palmar hyperhidrosis, anesthesia, botulinum toxin, cryoanalgesia, lidocaine, NRS scale

## Abstract

Primary palmar hyperhidrosis (PPH) constitutes a debilitating condition that profoundly impacts the social, functional, and occupational aspects of individuals. The intradermal administration of botulinum toxin type A (BoNT-A) stands as an established therapeutic approach for PPH, albeit one frequently accompanied by considerable pain, posing challenges for patient tolerance. Our study aimed to assess the efficacy of combining cryoanalgesia spray (CA) with topical anesthesia utilizing a cream containing liposomal lidocaine at a concentration of 40 mg/g, with the objective of mitigating the pain associated with intradermal BoNT-A injection for PPH treatment. Nineteen participants, aged ≥18 years and afflicted with severe PPH, were enrolled in a double-blind randomized vehicle-controlled trial. Patient-perceived pain during the procedure was quantified using the Numeric Rating Scale (NRS). Statistical analysis was applied to the collected data. The combination of CA and the topical application of liposomal lidocaine during BoNT-A treatment for PPH resulted in diminished pain compared to CA alone and the combination of CA with the application of a basic cream. Topical anesthesia through the application of a liposomal lidocaine-containing cream emerged as a facile, secure, and efficacious approach for alleviating the pain associated with intradermal BoNT-A injection in PPH treatment. Furthermore, it demonstrated compatibility with CA, thereby offering a comprehensive strategy for pain management during BoNT-A administration.

## 1. Introduction

Palmar hyperhidrosis (PH) is a relatively common condition characterized by excessive hand sweating beyond normal thermoregulatory needs [1]. Etiologically, the disorder can be primary (idiopathic) or secondary due to an underlying cause.

Primary palmar hyperhidrosis (PPH) typically begins in childhood, manifesting itself more strongly in ages of hormonal and sexual maturation during adolescence. An improvement after the fourth decade of life is common, and cases that persist after the fifth decade of life are rare [2].

The size or number of eccrine glands in PPH patients is not increased and these sweat glands do not possess any microscopic or macroscopic histopathologic abnormalities, suggesting a neurologic issue [3]. PPH may result from a complex malfunction of the autonomic nervous system involving both sympathetic and parasympathetic pathways, leading to the overstimulation of normal palmar eccrine sweat glands [3,4]. Studies on PPH patients have found altered cardiac autonomic function, marked by reduced reflex bradycardia after the Valsalva maneuver and increased vasoconstriction during cold-water finger immersion compared to controls, implying a potential link to broader autonomic dysfunction [5,6]. Another theory links hyperhidrosis to irregular central emotional regulation. Emotional and thermoregulatory sweating involves sympathetic cholinergic nerves, primarily regulated by the limbic system, anterior cingulate cortex, and hypothalamus. In PPH, it is proposed that the hypothalamic sweat center for palms operates solely under cortical control, potentially causing abnormal emotional sweating regulation [4,7].

While the exact cause of PPH remains intricate and not entirely understood, there seems to be a clear genetic component, suggesting autosomal dominant hereditary transmission with variable penetrance and no signs of X-linked inheritance [8]. The 14q11.2-q13 genetic locus was identified as being linked to palmar hyperhidrosis in Japanese individuals [9].

Symptoms are typically bilateral, symmetrical, and unaccompanied by other associated conditions. The palms exhibit cold, damp characteristics with a color range from pale to blush [10]. Sweating episodes have a sudden onset, whether triggered by emotional stress or not, with the most pronounced sweating occurring on the palms and fingers, and less so on the backs of the hands. Droplets form rapidly on the hands, and in some cases finger swelling may occur [11].

The diagnosis of PH is primarily clinical, based on medical history and physical examination. Key diagnostic criteria include visible, localized, and excessive sweating for at least six months, without an apparent cause, and meeting at least two of the following: bilateral and symmetrical sweat distribution, sweating at least once weekly, impairment in daily activities, onset before age 25, family history, and absence of sweating during sleep [3,12]. Plantar hyperhidrosis often co-occurs (57% of cases) with palmar hyperhidrosis [13]. PH can be confirmed through the Minor test, involving the application of a 2% alcoholic iodine solution to the test area, followed by the sprinkling of starch (e.g., cornstarch). The hyperhidrotic area solubilizes iodine, leading to a complexation reaction with starch. This interaction results in the evident development of dark blue staining [14].

A differential diagnosis is crucial to distinguish primary hyperhidrosis from associated conditions, which encompass endocrine issues (e.g., hyperthyroidism, diabetes), neurological factors (e.g., Parkinson’s disease, spinal cord injury), neoplastic conditions (e.g., CNS tumors, Hodgkin’s disease), infectious diseases (e.g., fever, tuberculosis), drugs, toxicity (e.g., alcoholism), and iatrogenic factors (e.g., postoperative sweating). All these conditions induce secondary generalized hyperhidrosis, unlike PH, which consists of localized sweating. Exceptions are spinal cord damage and reflex sympathetic dystrophy, possible causes of focal secondary hyperhidrosis [15,16].

Hyperhidrosis can profoundly affect one’s mental well-being, self-confidence, social engagement, personal relationships, and career decisions [17]. In clinical practice, a variety of questionnaires are employed to assess how hyperhidrosis influences one’s QoL (Quality of Life). The Hyperhidrosis Disease Severity Scale (HDSS), Hyperhidrosis Quality of Life Questionnaire (HQLQ), Dermatology Life Quality Index (DLQI), Keller questionnaire (Keller), and Campos Questionnaire (Campos) are the main validated questionnaires on the evaluation of severity and QoL for palmar hyperhidrosis [18]. Several studies in the literature have shown a high impact on quality of life in PH patients. Wolosker et al. found that 94% of 1658 patients with hyperhidrosis had poor or very poor QoL, and among the various forms of hyperhidrosis, PH was the most severe [19]. Dogru et al. found that among 165 PH patients, 38.2% had very poor QoL and 45.5% had poor QoL [20]. In a 2011 pre-post interventional study, 63.3% of PH patients had a very poor quality of life pre-sympathectomy [21]. A 2014 review describes the impact of primary hyperhidrosis on physical symptoms, i.e., axillary HH causes the staining of clothes and frequent bathing. PH reportedly affects manual activities and the handling of objects [22].

Therapy for PPH can be topical or systemic. Among the topical treatment options, the most significant are the use of astringents, iontophoresis, and botulinum toxin (BoNT). Systemic therapeutic options include anticholinergic drugs and psychotherapy. Video-assisted thoracoscopic sympathectomy (VATS) is an effective surgical approach for PPH [23].

Botulinum toxins (BoNTs), encompassing onabotulinum toxin A (Botox), abobotulinum toxin A (Dysport), incobotulinum toxin A (Xeomin), and rimabotulinum toxin B (Myobloc/Neurobloc), serve as primary or secondary treatments for primary palmar hyperhidrosis (PPH), exerting their effects by inhibiting acetylcholine release and impeding sweat secretion [24]. Despite their efficacy, BoNT therapy presents limitations, including contraindications in pregnancy and myasthenia gravis, off-label use for PPH, and potential interactions with other medications [25]. Administered through outpatient procedures, BoNT injections are evenly distributed across the palmar surface, with onabotulinum toxin A being the most commonly utilized variant at recommended doses of 100–150 units per hand [26]. Numerous studies affirm BoNTs’ effectiveness in managing PPH, with response rates ranging from 80 to 90% and effects lasting 6–9 months [27]. While complications such as injection site pain, swelling, and bruising are common, severe adverse events are infrequent [28]. Reported side effects encompass compensatory sweating, transient hand weakness, and palm dryness [29]. Additionally, repeated high-dose administrations may lead to complications like immunization with neutralizing antibodies, resulting in partial or complete clinical resistance [30].

Treatment-related pain is a major factor limiting the utility of BoNT in palmar surfaces; thus, administration in a pain-free environment is essential. Various anesthetic procedures exist for BoNT injection, with completely painless injections achievable through intravenous regional anesthesia (Bier’s block) or nerve blocks (ulnar, median, radial). In particular, compelling results have been achieved with the median nerve block in a recent study, demonstrating that local block significantly reduces pain during multiple injections of BoNT for treating focal HP. The study also recommends that the optimal site for local anesthesia injection should be located 4 cm distal to the transverse line of the pisiform, within the tendons of the palmaris longus and flexor carpi radialis muscles [31]. However, these methods are often poorly tolerated by patients and demand precision from the administering physician. Techniques like vibratory anesthesia and general anesthesia, while available, are impractical and lack robust scientific support. Innovative approaches such as needle-free anesthesia show promise but require further substantiation through additional studies before integration into clinical practice [32]. Moreover, these procedures may entail a high economic cost, limiting their widespread use. In current practice, cryoanalgesia and the application of topical anesthetics stand out as the most commonly employed analgesic methods for BoNT injection in primary palmar hyperhidrosis (PPH). Our study aimed to assess the clinical efficacy and safety of combining liposomal lidocaine with cryoanalgesia, comparing it with cryoanalgesia alone and cryoanalgesia combined with a control (basic cream) during BoNT-A treatment for PPH. We employed the Numeric Rating Scale (NRS), a widely used, statistically validated scale in clinical practice, to evaluate patients’ perceived pain during the injection procedure, with scores ranging from 0 to 10.

## 2. Results

Cryoanalgesia (CA) in combination with a topical application of liposomal lidocaine during the treatment of PPH with botulinum toxin A resulted in less pain compared with CA alone and the combination of CA and the application of basic cream.

### 2.1. Pain Scores according to the NRS Scale

**Figure 1:**
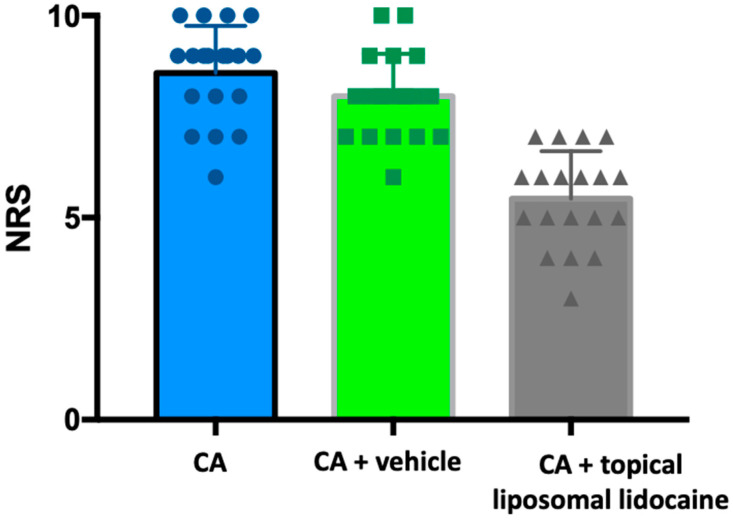
Pain scores in the patient subgroup. CA = cryoanesthesia.

The mean pain scores on the Numeric Rating Scale (NRS) for patients who underwent CA alone, CA in combination with basic cream, and CA in combination with anesthetic cream were 8.759 ± 1.17, 8 ± 1.50, and 5.47 ± 1.17, respectively (Figure 1). The medians were 9, 8 and 6, respectively. The maximum NRS value for CA+topical liposomal lidocaine was 7, compared with 10 for the maximum NRS for the other two groups; similarly, the minimum CA+topical liposomal lidocaine NRS value was 3, compared with 6 for the minimum NRS for the other two groups. The 25% and 75% percentiles were 8 and 9 for the CA group, 7 and 9 for the CA+ basic cream group, and 5 and 6 for the CA+ anesthetic cream group (Table 1).

It is noteworthy that the pain score for CA alone was found to be statistically higher than the score for CA combined with topical liposomal lidocaine (*p* < 0.01). Furthermore, the difference was also statistically significant in comparison with the control group (CA+basic cream) (*p* < 0.01) (Figure 1).

### 2.2. Safety Profile

No adverse events related to the application of the topical products described were observed, indicating their safety and tolerability by the study participants.

## 3. Discussion

The primary challenge associated with Botulinum Toxin (BoNT) injection for primary palmar hyperhidrosis (PPH) lies in the considerable pain experienced by patients during needle penetration into the densely innervated palm skin. Consequently, effective anesthetic procedures are crucial for pain management. Various anesthetic treatments are available to physicians performing BoNT injections for PPH.

Nerve blocks and Bier’s block are the most effective loco-regional anesthesia techniques for the injection treatment of PH, but they have some disadvantages. Nerve blocks are widely acknowledged as the primary anesthesia method for treating palmar and plantar hyperhidrosis (HH) [33]. Nevertheless, a significant number of healthcare practitioners either lack the ability or choose not to administer dependable nerve blocks. Additionally, a considerable portion of patients either refuse this form of anesthesia outright or are disinclined to undergo repeated nerve block procedures [34]. Drawbacks associated with nerve blocks encompass the risk of neural injury, vascular puncture, the temporary impairment of hand dexterity, lasting hours or days post-anesthesia, heightened susceptibility to bleeding at injection sites due to reactive hyperemia, and potential nerve scarring resulting from recurrent needle-induced injury [35]. General anesthesia is an overly invasive and time-consuming technique, and therefore not employable in clinical practice for pain control during the injection of BoNT [36]. Vibration anesthesia has been reported with inconsistent results [37]. Pressurized, needle-free “jet” injector devices such as Dermojet^®^ have been employed safely to inject BoNT-A itself directly into the dermis of the plantar HH without prior anesthesia [38]. The pain of injection was found to be tolerable in all patients treated with Dermojet^®^ [39]; however, the authors do not recommend its use on palmar skin because of potential injury to nerves or superficial palmar vessels. Naumann et al. compared BoNT-A injection with Dermojet^®^ versus needle injection and concluded that both techniques led to a significant reduction in hyperhidrosis. Nevertheless, needle injection was much more effective in controlling excessive sweating than the Dermojet^®^ technique [39]. A study showed that JetPeelTM-3, a medical device used for transdermal drug delivery by jet nebulization, can be used to administer lidocaine together with BoNT-A effectively and safely. However, the study is, to the best of our knowledge, unique in the literature and therefore the scientific evidence for this procedure is limited [40]. Cryoanalgesia and topical anesthetics are most likely the most frequently used anesthetic techniques in clinical practice.

Cryoanalgesia for primary palmar hyperhidrosis (PPH) can be administered using ice packs, dichlorotetrafluoroethane spray, or iced water. Kontochristopoulos et al. conducted a study comparing two cryoanalgesia techniques, dichlorotetrafluoroethane spray and ice packs, determining that dichlorotetrafluoroethane spray was more effective than ice packs [41]. However, a study by Lim et al. found that patients undergoing botulinum toxin injection for PPH were equally satisfied with cryoanalgesia using ice packs, dichlorotetrafluoroethane spray, and iced water [42]. Interestingly, one patient in the same study reported iced water cryoanalgesia to be more effective than either ethyl chloride spray or ice packs [43]. Comparative studies between cryoanalgesia and topical anesthetic application have been conducted, with one trial specifically evaluating these techniques in botulinum toxin injection for palmar hyperhidrosis. In this prospective study involving 23 patients, EMLA (Lidocaine/prilocaine) cream was applied to one palm, while ice was applied directly before injections in the other palm. The results demonstrated a 40% reduction in pain with ice application compared to EMLA cream [44].

Lidocaine cream, either alone or in combination with other local anesthetics like prilocaine, is beneficial for pain control during Botulinum Toxin (BoNT) injections, not only for primary palmar hyperhidrosis (PH) but also for other conditions such as dyskinesia. In a 2002 study involving 17 patients with facial dyskinesia undergoing BoNT injections for blepharospasm, pretreatment with EMLA cream (lidocaine/prilocaine) resulted in less painful injections compared to a placebo [45]. Beyond BoNT injections, lidocaine cream has demonstrated anesthetic efficacy in dermatology for various procedures. A combination of lidocaine 7% and tetracaine 7% in a cream vehicle has proven effective in controlling pain during laser therapies, including ablative and non-ablative laser resurfacing, laser hair removal, the laser treatment of vascular lesions, and laser tattoo removal [46,47]. In a study by Menter et al., the effectiveness of EMLA cream and 1% lidocaine infiltration in men for pain relief during the removal of genital warts by cryotherapy was analyzed. The application of lidocaine/prilocaine cream for 15 min significantly reduced the pain associated with lidocaine infiltration. Combining lidocaine/prilocaine cream with 1% lidocaine infiltration provided greater pain relief during cryotherapy compared to using either anesthetic alone [48].

A study by Bucalo et al. compared the anesthetic effects of liposomal lidocaine, non-liposomal lidocaine, and EMLA creams with a 30 min application time. The findings indicated that liposomal lidocaine cream exhibited a superior anesthetic effect compared to non-liposomal lidocaine. Furthermore, the study established the equivalence of efficacy between liposomal lidocaine 5% cream and EMLA cream [49]. In contrast, a recent randomized, intraindividual, comparative trial demonstrated that EMLA cream had a superior anesthetic effect compared to lidocaine 10% cream [50].

The current scientific literature on pain control in Botulinum Toxin (BoNT) injection for primary palmar hyperhidrosis (PPH) is both limited and conflicting, even for widely used techniques like anesthetic creams and cryoanalgesia. Consequently, our study addresses an existing gap in understanding, focusing on both cryoanalgesia and the concurrent use of anesthetic creams.

In our studied sample, cryoanalgesia performed via the spray technique demonstrated poor effectiveness (median NRS value of 9, mean of 8.579). The application of an occluded basic cream, while modestly improving Numeric Rating Scale (NRS) pain values, may be attributed to a placebo effect. Notably, combination anesthetic therapy involving cryoanalgesia and liposomal lidocaine cream proved highly effective in pain control during BoNT-A injection for PPH, yielding a median NRS value of 6 and a mean of 5.474.

Our study aligns with a lone case report in the literature, substantiating the findings with a larger sample size. This study of Patel et al. demonstrates a 75% decrease in patient-perceived pain during inactivation therapy with BoNT [51]. In our experience, spray cryoanalgesia alone proved inadequately effective. The combined use of spray cryoanalgesia and liposomal lidocaine 4% cream, considering limitations in clinical practice and the disparity in clinical evidence for various techniques, emerges as a promising strategy for pain control in BoNT-A injection for PPH. In addition, the study shows how the application of a liposomal lidocaine-containing cream emerged as a facile, secure, and efficacious approach for alleviating the pain associated with intradermal BoNT-A injection in PPH treatment.

Further studies are warranted to confirm our results, ideally through randomized, controlled, and comparative approaches. Exploring comparisons, such as liposomal lidocaine cryoanalgesia cream vs. EMLA cryoanalgesia cream, or testing different anesthetic creams with alternative cryoanalgesia techniques (e.g., ice packs or cold water), could provide valuable insights into the optimal combination for pain management in BoNT-A injection for PPH. Finally, further studies on the comparison of efficacy between lidocaine 4% cream, lidocaine 5% cream, and EMLA cream, even without cryoanalgesia in combination, would be desirable in our opinion, given the contradictory results of previous studies.

## 4. Materials and Methods

### 4.1. Study Design and Sample Characteristics

We performed a randomized double-blind controlled trial on patients aged ≥18 years. The study was conducted according to the Helsinki Declaration and after ethical approval from the Ethics Committee of Azienda Ospedaliero-Universitaria Ospedali Riuniti di Ancona (Protocol 2008 0710 OR on 28 January 2008). Informed consent was obtained from all subjects involved in the study.

We enrolled 19 patients (10 female and 9 male, mean age 39 ± 7 years) suffering from severe PPH resistant to antiperspirants containing aluminum chloride (resistance to treatment was defined as less than two points’ improvement in HDSS from baseline value).

All enrolled patients had already undergone BoNT-A treatment 6 months earlier with the sole aid of CA. On that occasion, the pain experienced during the treatment had been measured by a numeric rating scale (NRS), with a range from 0 to 10. In order to make the injection treatment less painful for the patient, we tested the efficacy of premedication with an anesthetic cream containing liposomal lidocaine (40 mg/g) (Asensil^®^ Logofarma Srl, Milan, Italy). A nurse applied 5 g of anesthetic cream on one of the two hands, in occlusive mode, by affixing a polyethylene film, while on the contralateral hand a vehicle base cream, also in occlusive mode, identical in color and texture, 45 min before the treatment was performed. Neither the physician performing the treatment nor the patient knew the site of application of the 2 creams. At the end of the treatment, which was nevertheless associated with cryoanalgesia, the patient was asked to indicate on an NRS scale the pain perceived by the hand during the treatment.

### 4.2. Botulin Toxin Type-A (BoNT-A)

**Figure 2:**
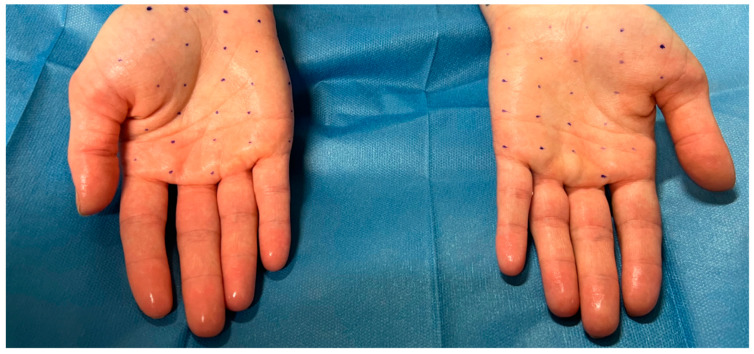
Patient suffering from severe palmar hyperhidrosis before carrying out the injection treatment. Note the shine of the hands caused by excessive sweating. Creation of the grids to define the injection points.

**Figure 3:**
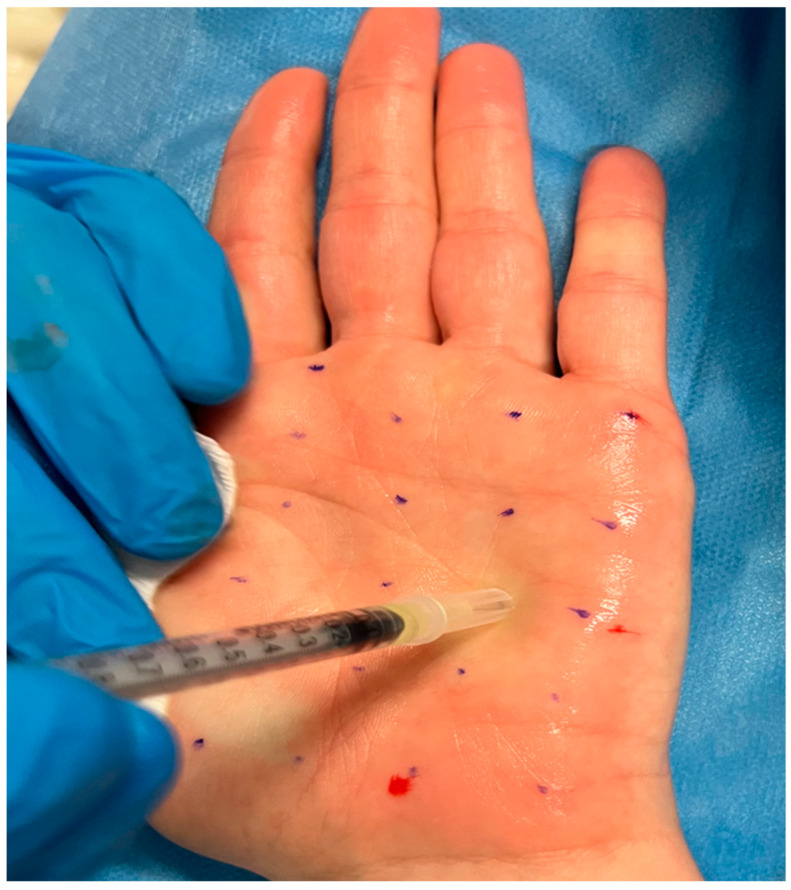
Injection treatment with BoNT-A.

By inactivating SNARE proteins at the presynaptic level, BoNT-A prevents the release of acetylcholine and other neurotransmitters that stimulate sweat secretion at the level of the eccrine gland [52]. After performing a guiding grid, we performed multiple intradermal injections of BoNT-A, appropriately diluted in saline at the palmar surface with a fine needle (30G), releasing 1 to 2 units of toxin depending on the sites. The number of injections performed per palm was between 20 and 25 for all patients. The distance between each injection was between 1 and 1,5 cm. The decision to administer this number of injections and at this distance was guided by both our clinical expertise and the current literature on the subject [33]. The procedure is illustrated in Figure 2 and Figure 3. BoNT-A injection is a safe treatment for PPH, with proven and long-lasting efficacy (the efficacy wears off in about 6 months). The main limiting factor in the treatment is the pain felt by the patient during the injection procedure. Cryoanalgesia can be performed for pain control.

### 4.3. Cryoanalgesia (CA)

Cryoanalgesia is a minimally invasive technique that exploits the pathophysiological principle that the application of cold results in conduction blockage, Wallerian degeneration, and predictable axonal regeneration over time, with the restoration of nerve function. Freezing of the target nerve branches thus causes a cessation of the conduction of pain impulses [53,54]. Cryoanalgesia in BoNT-A injection therapy for palmar hyperhidrosis can be performed with ice packs, dichlorotetrafluoroethane spray, or iced water [55]. We sprayed dichlorotetrafluoroethane at a distance of 2 to 4 inches for 3 to 4 s before each palmar injection. There was 1 to 2 s of frosting on the skin before the botulinum toxin was administered. We observed no adverse effects from cryoanalgesia spray in either injection therapy session.

### 4.4. Liposomal Lidocaine Cream

Lidocaine 40 mg/g cream is a local anesthetic for topical use for surface skin anesthesia prior to venous cannulation or venipuncture, or prior to the administration of painful topical treatments over larger areas of intact skin. Lidocaine alters depolarization in neurons by blocking sodium channels in the cell membrane. With an appropriate blockade, the membrane of the presynaptic neuron does not depolarize, preventing the action potential, thus giving an anesthetic effect [56]. Adequate anesthesia with lidocaine 4% cream can be achieved after 30 min of occlusive application, but the cream can be applied under a bandage for up to 5 h, except for infants aged 1 to 3 months, where it should not exceed 60 min, and infants aged 3 to 12 months, where it should not exceed 4 h [57]. The sample of patients we examined did not include infants. The amount of liposomal lidocaine cream applied was one fingertip unit (FTU) for the palm. The duration of occlusive dressing was 45 min. Local side effects of the drug may include irritation, redness, itching, or rash. Systemic side effects, such as anaphylactic shock, are rightly rare [57]. We observed no side or systemic effects from the application of lidocaine 4% occluded cream in our study.

### 4.5. NRS Scale

**Figure 4:**
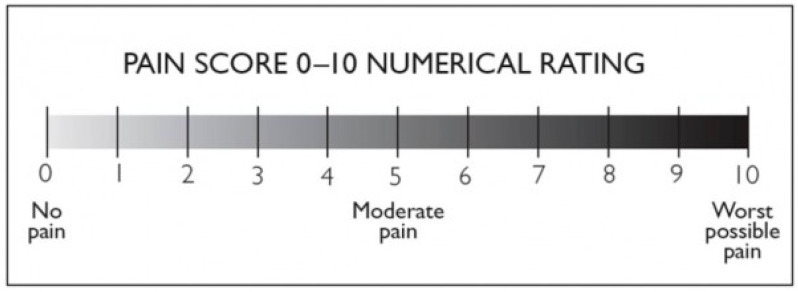
NRS pain scale with a range from 0 to 10.

The NRS scale is a one-dimensional 11-point scale that assesses pain intensity in adults [58]. The scale consists of a horizontal line, with a range from 0 to 10 corresponding to “no pain” and “worst pain imaginable,”, respectively. The patient indicates the intensity of his or her pain verbally or by drawing a circle on the number that best describes it [59]. Other 6, 7, 20, 21, and 101-point versions exist in the literature, but the 11-point version remains the most commonly used [59]. Compilation time is less than a minute and requires no visuomotor coordination, unlike the VAS scale. Test–retest reliability was good, but higher among literate patients (r = 0.96) than illiterate patients (r = 0.95). The minimum perceptible clinical improvement (MPCI) is 2 points or 33% [60]. The recruited patients quantified their perceived pain during the botulinum toxin injection procedure using an NRS scale with a range of 0 to 10, both when they performed cryoanalgesia alone and when they performed cryoanalgesia combined with anesthetic cream or control (basic cream). The group subjected solely to CA expressed overall perceived pain on both hands during the injective procedure. The group subjected to CA+vehicle expressed perceived pain at the level of the hand treated with vehicle. The group subjected to CA+topical liposomal lidocaine expressed perceived pain at the level of the hand treated with topical liposomal lidocaine. The values were recorded in an electronic medical record, then subjected to statistical analysis. The NRS scale we employed is shown in Figure 4.

### 4.6. Statistical Analysis

Graph-Pad Prism software (version 5.3, El Camino REAL, San Diego, CA, USA) was used to perform all statistical analyses. The collected data were analyzed with a Student’s t test, and a *p* value less than 0.05 was considered significant.

A limitation of our study is the small number of patients involved. Therefore, studies in larger cohorts of patients are required.

## Figures and Tables

**Table 1:** Results of statistical analysis.

Values	CA	CA+Vehicle	CA+Topical Liposomial Lidocaine
Number of values	19	19	19
Minimum	6	6	3
25% percentile	8	7	5
Median	9	8	6
75% percentile	9	9	6
Maximum	10	10	7
Mean	8.579	8	5.474
Std Deviation	1.17	1.054	1.172
Std. Error of Mean	0.2684	0.2418	0.2689
Lower 95% CI	8.015	7.492	4.909
Upper 95% CI	9.143	8.508	6.039

## Data Availability

Data are contained within the article.

## References

[1] Gregoriou S., Sidiropoulou P., Kontochristopoulos G., Rigopoulos D. (2019). Management Strategies of Palmar Hyperhidrosis: Challenges and Solutions. Clin. Cosmet. Investig. Dermatol..

[2] Strutton D.R., Kowalski J.W., Glaser D.A., Stang P.E. (2004). US prevalence of hyperhidrosis and impact on individuals with axillary hyperhidrosis: Results from a national survey. J. Am. Acad. Dermatol..

[3] Romero F.R., Haddad G.R., Miot H.A., Cataneo D.C. (2016). Palmar hyperhidrosis: Clinical, pathophysiological, diagnostic and therapeutic aspects. An. Bras. Dermatol..

[4] Nawrocki S., Cha J. (2019). The etiology, diagnosis, and management of hyperhidrosis: A comprehensive review: Etiology and clinical work-up. J. Am. Acad. Dermatol..

[5] Shih C.J., Wu J.J., Lin M.T. (1983). Autonomic dysfunction in palmar hyperhidrosis. J. Auton. Nerv. Syst..

[6] Birner P., Heinzl H., Schindl M., Pumprla J., Schnider P. (2000). Cardiac autonomic function in patients suffering from primary focal hyperhidrosis. Eur. Neurol..

[7] Glaser D.A., Hebert A.A., Pariser D.M., Solish N. (2007). Primary focal hyperhidrosis: Scope of the problem. Cutis.

[8] Ro K.M., Cantor R.M., Lange K.L., Ahn S.S. (2002). Palmar hyperhidrosis: Evidence of genetic transmission. J. Vasc. Surg..

[9] Higashimoto I., Yoshiura K., Hirakawa N., Higashimoto K., Soejima H., Totoki T., Mukai T., Niikawa N. (2006). Primary palmar hyperhidrosis locus maps to 14q11.2-q13. Am. J. Med. Genet. Part A.

[10] Hasimoto E.N., Cataneo D.C., Reis T.A.D., Cataneo A.J.M. (2018). Hyperhidrosis: Prevalence and impact on quality of life. J. Bras. Pneumol..

[11] Murray C.A., Cohen J.L., Solish N. (2007). Treatment of focal hyperhidrosis. J. Cutan. Med. Surg..

[12] Haider A., Solish N. (2005). Focal hyperhidrosis: Diagnosis and management. CMAJ.

[13] Vetrugno R., Liguori R., Cortelli P., Montagna P. (2003). Sympathetic skin response: Basical mechanisms and clinical applications. Clin. Auton. Res..

[14] Muller S.A., Kierland R.R. (1959). The use of a modified starch-iodine test for investigating local sweating responses to intradermal injection of methacholine. J. Investig. Dermatol..

[15] Eisenach J.H., Atkinson J.L., Fealey R.D. (2005). Hyperhidrosis: Evolving therapies for a well-established phenomenon. Mayo Clin. Proc..

[16] Leung A.K., Chan P.Y., Choi M.C. (1999). Hyperhidrosis. Int. J. Dermatol..

[17] Lenefsky M., Rice Z.P. (2018). Hyperhidrosis and its impact on those living with it. Am. J. Manag. Care.

[18] Parashar K., Adlam T., Potts G. (2023). The Impact of Hyperhidrosis on Quality of Life: A Review of the Literature. Am. J. Clin. Dermatol..

[19] Wolosker N., Kauffman P., de Campos J.R.M., Faustino C.B., da Silva M.F.A., Teivelis M.P., Puech-Leão P. (2020). Long-term results of the treatment of primary hyperhidrosis with oxybutynin: Follow-up of 1658 cases. Int. J. Dermatol..

[20] Dogru M.V., Sezen C.B., Girgin O., Cansever L., Kocaturk C.I., Metin M., Dincer S.I. (2020). Is there any relationship between quality of life and the level of sympathectomy in primary palmar hyperhidrosis? Single-center experience. Gen. Thorac. Cardiovasc. Surg..

[21] Wang F.G., Chen Y.B., Yang W.T., Shi L. (2011). Comparison of compensatory sweating and quality of life following thoracic sympathetic block for palmar hyperhidrosis: Electrocautery hook versus titanium clip. Chin. Med. J..

[22] Hamm H. (2014). Impact of hyperhidrosis on quality of life and its assessment. Dermatol. Clin..

[23] Pariser D.M., Ballard A. (2014). Iontophoresis for palmar and plantar hyperhidrosis. Dermatol. Clin..

[24] Samizadeh S., De Boulle K. (2018). Botulinum neurotoxin formulations: Overcoming the confusion. Clin. Cosmet. Investig. Dermatol..

[25] Hosp C., Hamm H. (2017). Safety of available and emerging drug therapies for hyperhidrosis. Expert Opin. Drug Saf..

[26] Solish N., Bertucci V., Dansereau A., Hong H.C.-H., Lynde C., Lupin M., Smith K.C., Storwick G. (2007). A comprehensive approach to the recognition, diagnosis, and severity-based treatment of focal hyperhidrosis: Recommendations of the Canadian Hyperhidrosis Advisory Committee. Dermatol. Surg..

[27] Campanati A., Giuliodori K., Martina E., Giuliano A., Ganzetti G., Offidani A. (2014). Onabotulinumtoxin type A (Botox^®^) versus Incobotulinumtoxin type A (Xeomin^®^) in the treatment of focal idiopathic palmar hyperhidrosis: Results of a comparative double-blind clinical trial. J. Neural Transm..

[28] Mannava S., Mannava K.A., Nazir O.F., Plate J.F., Smith B.P., Koman L.A., Tuohy C.J. (2013). Treatment of palmar hyperhidrosis with botulinum neurotoxin a. J. Hand Surg..

[29] Gregoriou S., Rigopoulos D., Makris M., Liakou A., Agiosofitou E., Stefanaki C., Kontochristopoulos G. (2010). Effects of botulinum toxin—A therapy for palmar hyperhidrosis in plantar sweat production. Dermatol. Surg..

[30] Basciani M., Di Rienzo F., Bizzarrini M., Zanchi M., Copetti M., Intiso D. (2014). Efficacy of botulinum toxin type B for the treatment of primary palmar hyperhidrosis: A prospective, open, single-blind, multi-centre study. Arch. Dermatol. Res..

[31] Yi K.H., Lee J.H., Hu H., Kim J.H., Park H.J., Kim K.B., Kim J.H., Kim H.J. (2023). Anatomical proposal of local anesthesia injection for median nerve block in treating hyperhidrosis with botulinum neurotoxin. Surg. Radiol. Anat..

[32] Benohanian A. (2009). What stands in the way of treating palmar hyperhidrosis as effectively as axillary hyperhidrosis with botulinum toxin type A. Dermatol. Online J..

[33] Glaser D.A., Hebert A.A., Pariser D.M., Solish N. (2007). Palmar and plantar hyperhidrosis: Best practice recommendations and special considerations. Cutis.

[34] Smith K.C., Comite S.L., Storwick G.S. (2007). Ice minimizes discomfort associated with injection of botulinum toxin type a for the treatment of palmar and plantar hyperhidrosis. Dermatol. Surg..

[35] Hayton M., Stanley J., Lowe N. (2003). A review of peripheral nerve blockade as local anaesthesia in the treatment of palmar hyperhidrosis. Br. J. Dermatol..

[36] Locke M.C., Davis J.C., Brothers R.J., Love W.E. (2018). Assessing the outcomes, risks, and costs of local versus general anesthesia: A review with implications for cutaneous surgery. J. Am. Acad. Dermatol..

[37] Saijo M., Ito E., Ichinohe T., Kaneko Y. (2005). Lack of pain reduction by a vibrating local anesthetic attachment: A pilot study. Anesth. Prog..

[38] Vadoud-Seyedi J. (2004). Treatment of plantar hyperhidrosis with botulinum toxin type A. Int. J. Dermatol..

[39] Naumann M., Bergmann I., Hofmann U., Hamm H., Reiners K. (1998). Botulinum toxin for focal hyperhidrosis: Technical considerations and improvements in application. Br. J. Dermatol..

[40] Iannitti T., Di Cerbo A., Aspiro A., Palmieri B. (2014). A preliminary study of painless and effective transdermal botulinum toxin A delivery by jet nebulization for treatment of primary hyperhidrosis. Drug Des. Devel. Ther..

[41] Kontochristopoulos G., Gregoriou S., Zakopoulou N., Rigopoulos D. (2006). Letter: Cryoanalgesia with Dichlorotetrafluoroethane Spray Versus Ice Packs in Patients Treated with Botulinum Toxin-A for Palmar Hyperhidrosis: Self-Controlled Study. Dermatol. Surg..

[42] Lim E.C., Seet R.C. (2007). Another Injection-free method to effect analgesia when injecting botulinum toxin for palmar hyperhidrosis: Cryoanalgesia. Dermatol. Online J..

[43] Lim E.C., Seet R.C. (2006). Physician, treat thyself. BMJ.

[44] Alsantali A. (2018). A comparative trial of ice application versus EMLA cream in alleviation of pain during botulinum toxin injections for palmar hyperhidrosis. Clin. Cosmet. Investig. Dermatol..

[45] Söylev M.F., Koçak N., Kuvaki B., Özkan S.B., Kir E. (2002). Anesthesia with EMLA cream for botulinum A toxin injection into eyelids. Ophthalmologica.

[46] Cohen J.L. (2013). Pain management with a topical lidocaine and tetracaine 7%/7% cream with laser dermatologic procedures. J. Drugs Dermatol..

[47] Greveling K., Prens E.P., Ten Bosch N., van Doorn M.B. (2017). Comparison of lidocaine/tetracaine cream and lidocaine/prilocaine cream for local anaesthesia during laser treatment of acne keloidalis nuchae and tattoo removal: Results of two randomized controlled trials. Br. J. Dermatol..

[48] Menter A., Black-Noller G., Riendeau L.A., Monti K.L. (1997). The use of EMLA cream and 1% lidocaine infiltration in men for relief of pain associated with the removal of genital warts by cryotherapy. J. Am. Acad. Dermatol..

[49] Bucalo B.D., Mirikitani E.J., Moy R.L. (1998). Comparison of skin anesthetic effect of liposomal lidocaine, nonliposomal lidocaine, and EMLA using 30-minute application time. Dermatol. Surg..

[50] Junputipong N., Rojhirunsakool S., Deewongkij P., Kamanamool N., Udompataikul M. (2022). Comparison of the onset, depth, and duration of cutaneous anesthesia between topical 10% lidocaine and EMLA creams: A randomized, intraindividual, comparative trial. J. Dermatol. Treat..

[51] Patel R., Halem M., Zaiac M. (2009). The combined use of forced cold air and topical anesthetic cream for analgesia during the treatment of palmar hyperhydrosis with botulinum toxin injections. J. Drugs Dermatol..

[52] Martina E., Diotallevi F., Radi G., Campanati A., Offidani A. (2021). Therapeutic Use of Botulinum Neurotoxins in Dermatology: Systematic Review. Toxins.

[53] Moorjani N., Zhao F., Tian Y., Liang C., Kaluba J., Maiwand M.O. (2001). Effects of cryoanalgesia on post-thoracotomy pain and on the structure of intercostal nerves: A human prospectiverandomized trial and a histological study. Eur. J. Cardiothorac. Surg..

[54] Filippiadis D., Efthymiou E., Tsochatzis A., Kelekis A., Prologo J. (2021). Percutaneous cryoanalgesia for pain palliation: Current status and future trends. Diagn. Interv. Imaging.

[55] Ernst E., Fialka V. (1994). Ice freezes pain? A review of the clinical effectiveness of analgesic cold therapy. J. Pain Symptom Manage..

[56] Hermanns H., Hollmann M.W., Stevens M.F., Lirk P., Brandenburger T., Piegeler T., Werdehausen R. (2019). Molecular mechanisms of action of systemic lidocaine in acute and chronic pain: A narrative review. Br. J. Anaesth..

[57] ASENSIL (lidocaine) [Prescribing Information]. https://farmaci.agenziafarmaco.gov.it/aifa/servlet/PdfDownloadServlet?pdfFileName=footer_003674_043742_RCP.pdf&sys=m0b1l3.

[58] Childs J.D., Piva S.R., Fritz J.M. (2005). Responsiveness of the numeric pain rating scale in patients with low back pain. Spine.

[59] Williamson A., Hoggart B. (2005). Pain: A review of three commonly used pain rating scales. J. Clin. Nurs..

[60] Hawker G.A., Mian S., Kendzerska T., French M. (2011). Measures of adult pain: Visual Analog Scale for Pain (VAS Pain), Numeric Rating Scale for Pain (NRS Pain), McGill Pain Questionnaire (MPQ), Short-Form McGill Pain Questionnaire (SF-MPQ), Chronic Pain Grade Scale (CPGS), Short Form-36 Bodily Pain Scale (SF-36 BPS), and Measure of Intermittent and Constant Osteoarthritis Pain (ICOAP). Arthr. Care Res..

